# Modelling the effect of religion on human empathy based on an adaptive temporal–causal network model

**DOI:** 10.1186/s40649-017-0049-z

**Published:** 2018-01-05

**Authors:** Laila van Ments, Peter Roelofsma, Jan Treur

**Affiliations:** 1 Behavioural Informatics Group, Department of Computer Science, Vrije Universiteit Amsterdam, De Boelelaan 1081, 1081 HV Amsterdam, The Netherlands; 2 Theological Seminary for International Churches and Ministries, Theological University Kampen, Broederweg 15, 8261 GS Kampen, The Netherlands

## Abstract

**Background:**

Religion is a central aspect of many individuals’ lives around the world, and its influence on human behaviour has been extensively studied from many different perspectives.

**Methods:**

The current study integrates a number of these perspectives into one adaptive temporal–causal network model describing the mental states involved, their mutual relations, and the adaptation of some of these relations over time due to learning.

**Results:**

By first developing a conceptual representation of a network model based on the literature, and then formalizing this model into a numerical representation, simulations can be done for almost any kind of religion and person, showing different behaviours for persons with different religious backgrounds and characters. The focus was mainly on the influence of religion on human empathy and dis-empathy, a topic very relevant today.

**Conclusions:**

The developed model could be valuable for many uses, involving support for a better understanding, and even prediction, of the behaviour of religious individuals. It is illustrated for a number of different scenarios based on different characteristics of the persons and of the religion.

## Background

Religion is a topic that every person has an opinion about, whether that opinion is positive or negative. While some people blame religion for war and terrorism, others believe that religion is the only bright spot in a world full of mischief. Does religion cause individuals to be more empathic, enabling them to be aware of the others’ feelings, needs and wants? Or, is religion a cause for human dis-empathy, making persons indifferent or even hostile for their fellow human? A clear answer has not yet been found, even though a lot of research has been done on the topic; e.g. [28, 31, 43, 51]. Questioning the influence of religion on human behaviour may not deserve a yes or no type of answer, but rather an answer that involves more aspects such as one’s character, culture, and different kinds of religions. In some way, all aspects and influences indicated above come together and originate in the brain. A lot of research has been done on how human behaviour is generated in the brain, also concerning religious topics. So, if these processes in the brain related to religion can be represented, this could help to get an answer to the question.

A method that can be used to represent real world processes concerning human beings is Network-Oriented Modelling. By this method, mechanisms that are based on neurological mechanisms are represented in a network model using different states and connections between them, as described in [47, 48]. This Network-Oriented Modelling method can be used to simulate behaviour of individuals with different religious backgrounds, characters and cultures.

In this paper, first, in “Literature overview” section, a brief literature overview on the existing research related to the topic is discussed. Then, in “The temporal–causal network model” section the conceptual representation of the network model with its various parts is discussed, and it is indicated how a numerical formalization of this model was obtained. In “Simulation scenarios” section, a relevant scenario simulated using the model is discussed; “Discussion and conclusion” is a discussion.

## Literature overview

There are two important approaches that are used to explain the origins of religion and religion-based behaviour. First, there is the evolutionary approach [3, 9, 11, 46] that tries to explain the origin and different aspects of religion from an evolutionary perspective. Second, there is the neurotheologist approach [5, 12, 41] that tries to find the origins of religion in the brain and explain religious behaviour on the basis of neurological processes. Further scientific and philosophical developments from both different perspectives around cognition, neuroscience and conscious thinking will most likely generate useful insights into religion [51]. Therefore, an approach that combines these different aspects into one model would give the most promising answer to our question. Such a kind of multidisciplinary model is indicated in two articles by Kapogiannis et al. [27, 28], proposing an integrative cognitive neuroscience framework for understanding the cognitive and neural foundations of religions. Among others using MRI analysis, they define three dimensions that together form an individual’s religious belief. The first one is God’s perceived level of involvement, the second God’s perceived level of emotion, and finally the doctrinal and experiential religious knowledge of an individual. Kapogiannis et al. considered these dimensions as nodes of a network and examined the causal flow within and between such networks, together forming the individual’s religious belief. Also, some other studies on religion have been combining knowledge from multiple disciplines, such as [43, 54], although the distinction between the different perspectives on religion was still kept.

Besides the above described approaches to religion, many experiments have been done to examine behaviour of religious persons. As explored by [31], religion can foster implicit self-regulation among religious individuals, unconsciously changing their actions and regulating their emotions. Also, religious individuals that prayed for people that angered them showed less aggression towards those people afterwards, indicating that religious behaviour can change people’s emotions [10]. Furthermore, a study of Schjoedt et al. [44] found that praying towards God activates brain regions that are responsible for active interpersonal interactions and enable people to generate an internal representation about ‘the other’, in this case God. This proves that praying individuals consider God a real meaningful person, rather than a fictive or abstract entity. This idea of internally representing God as a person is also discussed in [46].

Regarding this theory of God as a real meaningful person, an interesting idea can be developed as follows. As described in [33, 47, 48], a person can develop an empathic understanding of others through mirroring and internal simulation mechanisms, and these mechanisms also influence the individual beliefs and actions of that person. As a result, the aforementioned internal representation which individuals generate when they communicate with God, as a real meaningful person, can also generate an empathic understanding of God as perceived by the individual. This way, the individual mirrors the (internally represented) beliefs, actions and emotions of their perceived God. The combination of these mechanisms enables the image that an individual has of God to influence his own beliefs, actions and emotions, in a way similar to how an individual is influenced by other humans. The image that an individual has of God (e.g. ‘the God-image’ which will be described more extensively later on), and how this image has impact on the individual, can involve many aspects. One example is studied by Granqvist et al. [22], who examined the God-image as an attachment figure in theistic religions, defining the relationship with God as an attachment relationship. Granqvist et al. examine the influence of a person’s attachment style to the person’s relationship to God. Another example that was studied is the impact of the character that an individual’s God-image has. For example, an individual whose God-image is based on an authoritarian figure (like ‘God is great’, or ‘God strikes down in anger’) act in more antisocial, dis-empathic ways, and believers whose attachment relationship with God is a loving one (‘God is love’) are acting in a more social, empathic manner [16, 26, 39]. Finally, there is an influence of the level of judgmentalism in a person’s God image on the willingness to volunteer both in internal and external communities [34]. However, as described above, the influence of religion on human empathy and dis-empathy does not emerge from one single input, but from the combination of the individual’s character and his God-image, which are both (partly) formed by the individual’s experiences and knowledge.

## The temporal–causal network model

**Fig. 1:**
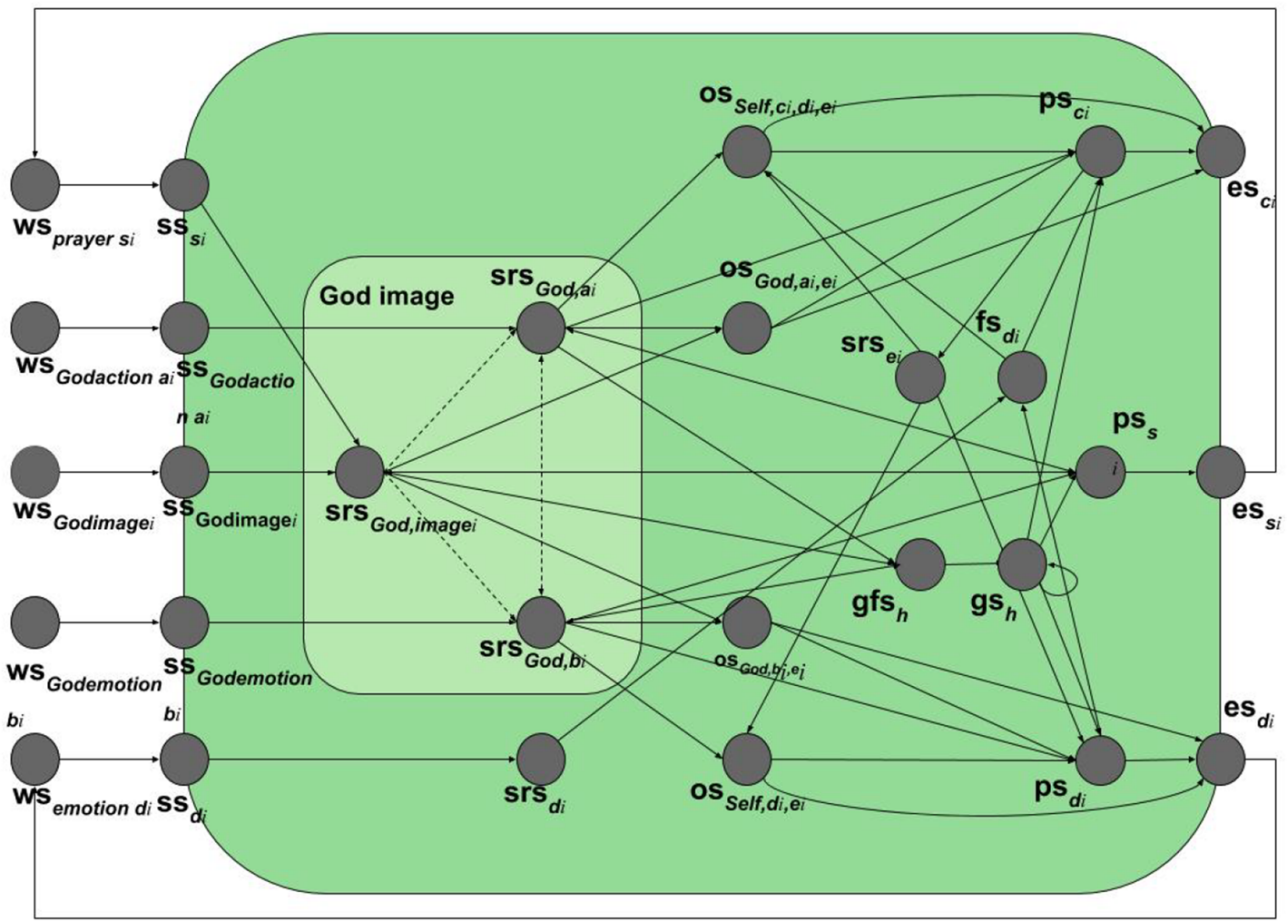
Graphical conceptual representation of the temporal–causal network model; here subscript i denotes either empathy (1) or dis-empathy (2)

In this section, it is presented how a neurologically inspired network model can be made that simulates the influence of religion on an individual’s (dis)empathic behaviour and emotions towards others. The model was developed according to the Network-Oriented Modelling approach based on temporal–causal networks described in [47, 48] and adopts elements of previously developed network models for joint decision making processes [33] and action ownership [49]. It is based on different theories on religion and human behaviour from the literature which will be explained below. Combining these, an integrative computational network model was created that focuses on the influence of religion on (dis)empathic behaviour and emotions towards others. First, “Mirror neurons and internal simulation”, “Action ownership states for God and Self”, and “The God-image” sections present how theories and literature were used to construct the model, leading to a conceptual representation of the network model depicted in Fig. 1. Then, “From conceptual to numerical representation of the model” section explains how a numerical representation was obtained from this conceptual representation.

### Mirror neurons and internal simulation

Mirror neurons enable sensory input, for example, an observed action or body state of another person, to directly affect a person’s own preparation state. In the current model, this is modelled by direct links from the sensory representation states of the emotions and actions of the God-image to the preparation states for emotions and behaviour of the Self. This gives the preparation state a similar function as a mirror neuron has: it becomes active after observing the action or the emotion. This mirror neuron function of preparation states makes that the actions and emotions of the God-image affect the corresponding behaviour, emotion and prayer states of the Self, leading to the actions and emotions of the God-image to influence the behaviour, emotions and prayers of the Self. The mirror neuron function enables to influence the individual’s own preparation states. Then, due to activation of the preparation states, the actions or emotions are internally simulated in a process as described by William James [15] and Antonio Damasio [8, 9]; this involves the following process. A world state ws_*W*_, a situation *W* in the world, occurs representing another person’s action or emotion expression *X*. The person develops a sensory state ss_*W*_ of this world state, and then a sensory representation state srs_*W*_ of it. Now by its mirror neuron function the preparation state ps_*X*_ for bodily changes for the same action or emotion *X* occurs. Depending on the context, this is expressed or executed, indicated by state es_*X*_. Execution of an action is modelled by an *action execution loop* and the process involving expression of an emotion by a *body loop*. In the model, the body loop is modelled by the link from an individual’s execution state of a body state expressing an emotion to the individual’s sensory representation of that body state. The feeling for the emotion is based on this sensory representation of the body state. However, the process is extended by adding a possibility by internal simulation without executing an actual action (as-if body loop). This process is incorporated in the model by a (predictive) loop from the preparation state for an action or emotion to the sensory representation for its effect, enabling direct emotion formation without behaviour execution.

### Action ownership states for God and Self

Whether an individual performs certain behaviour or expresses emotions that were mirrored (e.g. from the God-image) depends on the context. This context is represented by action ownership states for which a model was introduced in [49]. An ownership state is an indication to what extent an individual attributes an action or emotion to himself, or to what extent the individual deems someone else responsible. This ownership state for an action (which can also apply to an emotional response) can lead to a ‘go’ or ‘no-go’ decision for behaviour or emotion expression. There are four different ownership states in the model; see Table 1.

**Table 1:** Ownership states for God and Self for actions and emotions

	God-ownership state	Self-ownership state
Action	${\text{os}}_{{God,a_{i} ,e_{i} ,b_{i} }}$	${\text{os}}_{{Self,c_{i} ,e_{i} ,b_{i} }}$
Emotion	${\text{os}}_{{God,b{}_{i},e_{i} }}$	${\text{os}}_{{Self,b{}_{i},e_{i} }}$

Here ${\text{os}}_{{Self,c_{i} ,e_{i} ,b_{i} }}$ is the Self-ownership state for behaviour *c*_*i*_ with predicted effect *e*_*i*_ and related feeling *b*_*i*_. It is influenced by the sensory representation state ${\text{srs}}_{{God,a_{i} }}$ of God performing action *a*_*i*_, the sensory representation of *e*_*i*_ and the feeling state for *b*_*i*_. In turn, it influences both the preparation state ${\text{ps}}_{{c_{i} }}$ for that behaviour *c*_*i*_ and the execution state ${\text{es}}_{{c_{i} }}$ for that behaviour. Furthermore, God-ownership ${\text{os}}_{{God,a_{i} ,e_{i} ,b_{i} }}$ for action *a*_*i*_ is influenced by the sensory representation states ${\text{srs}}_{{God,a_{i} }}$ and ${\text{srs}}_{{God,image_{i} }}$ of the God-image. In turn, by mirroring it affects the preparation state ${\text{ps}}_{{c_{i} }}$ for the related behaviour of Self and the execution state ${\text{es}}_{{c_{i} }}$ for that behaviour. Moreover, Self-ownership ${\text{os}}_{{Self,b{}_{i},e_{i} }}$ of emotional response *b*_*i*_ related to *e*_*i*_ is influenced by the sensory representation ${\text{srs}}_{{God,b_{i} }}$ of the emotion within the God-image and the person’s sensory representation ${\text{srs}}_{{e_{i} }}$ of the predicted effect *e*_*i*_. In turn, ${\text{os}}_{{Self,b{}_{i},e_{i} }}$ influences the preparation state ${\text{ps}}_{{b_{i} }}$ of the emotion and the execution state ${\text{es}}_{{b_{i} }}$ (expression) of the emotion *b*_*i*_. Finally, God-ownership state ${\text{os}}_{{God,b{}_{i},e_{i} }}$ of emotional response *b*_*i*_ related to *e*_*i*_ is influenced by the sensory representation states ${\text{srs}}_{{God,b_{i} }}$ of God’s emotion and ${\text{srs}}_{{God,image_{i} }}$ of the God-image. In turn, it affects the preparation state ${\text{ps}}_{{d_{i} }}$ for the related emotion *d*_*i*_ and the execution state ${\text{es}}_{{d_{i} }}$ for that emotion. With the distinction between the ownership of God over behaviour and emotions that the individual expresses, the level of involvement and authority of God that an individual experiences is represented, as brought forward in [27]. An individual with a very low Self-ownership and a high God-ownership can show behaviour different from an individual with a high Self-ownership and a low God ownership.

### The God-image

The notion of the God-image has received a lot of attention in the scientific world in the past years, studying the influence of this phenomenon, and more specifically its influence on human behaviour towards others [26, 34, 39]. Different kinds of God-images have proved to influence human behaviour towards others in different ways. For example, where an authoritarian, punishing and controlling God-image is correlated to aggressive, dis-empathic behaviour, a forgiving, helping God-images correlates to prosocial, empathic behaviour [26, 34]. Furthermore, the belief in Godly omnipresence and omnipotence also influences human prosociality: individuals with a moralistic, all knowing God-image showed more prosocial behaviour than individuals with a non-moral or non-all-knowing God-image [39].

Besides the studies on the influence of the God-image on human behaviour towards others, this process can also be described from the mentalizing perspective, as introduced by Schaap-Jonker [43]. Mentalizing is the capacity of thinking about thinking and feeling. It provides awareness that one’s own and others’ behaviour is driven by mental states, and gives the ability to selectively activate internal states that fit the individual’s particular. Also, mentalizing generates a subjective experience of agency, this way supporting a sense of identity [1, 2, 18, 43]. Mentalizing also bears some resemblance to the process of internal simulation as described in [47], where an individual internally simulates mind states to predict effects in the external world or other persons. Mentalizing can occur both consciously or unconsciously, concern the self or others, and is both cognitive and affective [19]. This creates many possibilities in the interactions of the individual towards the God-image.

To enable a God-image to influence an individual’s behaviour as explained above, the individual first has to have a God-image. Below, the inputs that generate the God-image are described. The God-image refers to the personal God of the individual. As discussed in [27, 28, 42], this God-image consists of both an emotional part and a cognitive part, and both parts are dynamically interrelated. The emotional part is unconsciously developed, highly influenced by parents and significant others, as discussed in “Input from religious texts” section. The cognitive part of the God-image consists of the knowledge an individual has about God, like the doctrinal information the individual received in religious study, at school, or at church, see “Input from parents or other caregivers” section. Finally, an input that brings together the emotional and cognitive part of the God-image is prayer, or active communication with God, as explained in “Input from prayer” section. The emotional and the cognitive part that form the God-image can be traced back to different parts in the brain as studied by [27, 28, 42]. The emotional part involves the amygdala, basal ganglia, the ventromedial prefrontal cortex, the lateral temporal cortex, the dorsal anterior cingulated cortex and the orbitofrontal cortex. These parts of the brain are involved in assigning emotional significance to behaviour and events and to controlling cognition and emotion. On the other hand, the cognitive part involves the lateral prefrontal cortex, the medial prefrontal cortex, the lateral parietal cortex, the medial parietal cortex and the medial temporal lobe, all brain circuits that are responsible for the processing of more complex linguistic and symbolic input. This combination of brain processes results in the formation of the personal God-image of the individual; each personal God-image differs based on the individual’s personal character, experiences and knowledge, which will be discussed more extensively below.

#### Input from religious texts

Reading religious scriptures or prayers where the interaction between God and humans play an essential role activates brain regions involved in personal interaction [10, 44]. Therefore, reading these scriptures can influence the God image of (religious) individuals, and also the individual’s actions towards others [20], making religious scriptures an important basis for the God image and behaviour of an individual. Or as phrased by Metzger and Coogan ([35], p. vi):

‘The Bible frames the essential principles of how human beings should deal with God and with each other’

This is a statement that can also be extended to other religious scriptures. A lot of research has been done on this relation between religious scriptures, God image and human behaviour [38, 52–54]. This section explores some similarities and differences between the representation of the God image in religious sources between religions, namely the Christian Bible, Torah and Qur’an, and their possible influence on the model.

First of all, there is a theme that seems to be playing an important role in religious texts of various religions, namely that of awe. This can be considered an expected result of the fact that awe strengthens belief and commitment to religion, as was studied by [40, 52]. In Christianity, Judaism and Islam awe is a reoccurring, central aspect of important religious texts. For example in the first sentence of the legal work of Maimonides, one of the most important scholars in Judaism:

‘The foundation of all foundations and the pillar of wisdom is to know that there is a Primary Being who brought into being all existence’

(Laws of the Foundation of the Torah 1:1, Moznaim Trans.), in the Christian Bible (John 6:29), and in the first of the five pillars of Iman, faith, of Islam.

Another similarity among religious texts is the differentiation between in-groups and out-groups’ religions, found in the Bible, Torah and Qur’an. Analysis of the scriptures shows that the relation between God and the in-group is characterized by words such as helpful, loving, while the relation between God and the out-group is more characterized by hurting, oppose, bad. However, these differences were more directed to the positive side for in-group narratives, as opposed to a negative approach towards out-groups, suggesting the importance of positive psychological mechanisms in these scriptures and their religions [54].

There are also slight differences between the three religions, although the researchers mention that this can depend on the translation used or the specific part of the scripture that is highlighted [38, 53, 54]. Another interesting point comes forward in the study of Weingarten et al. [54], where in an analysis of scripture the researchers looked at the frequent appearance of Godly divinity in the scriptures in the combination with “humble” cluster themes, which encompass being humble, grateful, trusting, and to pray/appeal. This could be because the humility or submissiveness of an individual is triggered by their relationship with the divine God image.

The extent of divinity of the God-image between the three religions discussed does has a difference in Weingarten’s research. Among some other differences, the Torah and New Testament both had global top ranking global themes for God’s ‘help’ and ‘cooperate’, while the Qur’an which had ‘divine’ W and ‘oppose’ as global top ranking themes. This last difference could have an interesting connection to religious fundamentalism. According to a study on fundamentalists’ attitudes toward outgroups as a function of exposure to authoritative religious texts [8], submissiveness to religious authority is one of the main factors in the generation of religious fundamentalism. The fact that Godly divinity is the top ranking global theme in the Qur’an could lead among a higher level of submissiveness. Also, the study found that fundamentalism causes low prosocial attitudes toward out-groups after exposure to violent biblical texts, and high prosocial attitudes toward in-groups after exposure to positive religious texts, while this effect of religious text on pro- or antisocial behaviour did not apply to religious individuals in general (although partially with positive religious text increasing prosocial behaviour).

So, although religious fundamentalists exist in every religion, the higher occurrence of Godly divinity based in the Qur’an can be a factor in the generation of religious fundamentalism. For example, when a person has both an anxious attachment relationship to God, see the section below, which is often an all-consuming and clingy relationship, and a lot of influence from Qur’an scriptures, this could be a basis for religious fundamentalism. However, many other factors are also important in this process.

So, how would this discussed theory influence the behaviour of the developed computational model? The influence of the different scriptures mostly lies in the World States, namely ws_*God_image*_, ${\text{ws}}_{{God\_action\_a_{i} }}$ and ${\text{ws}}_{{God\_emotion\_b_{i} }}$, this way the God image is influenced either by empathic or dis-empathic influences. Also, the divinity of God can be expressed in the ownership states: if the relationship between an individual and God is one between the submissive and the divine, the ownership of the individual is relatively low in comparison to the God ownership.

#### Input from parents or other caregivers

The relationship to God is an essential aspect of one’s personal religion. This relationship between an individual and God closely resembles an attachment relationship in various ways: proximity maintenance, God as a safe haven, God as a real person and God as a stronger and wiser entity [13, 22–24, 37]. First of all, proximity maintenance, a central aspect of the attachment theory, is present in religion in the attempt to become closer to God. For example, praying individuals often strive to become closer to God, and being separated from God is the opposite of what a religious individual wants [23].

Second, God is often perceived as a secure base, and people are likely to turn to God during periods of stress [10], and research showed that God’s traits that are mentioned most often by believers are loving, supportive, guiding, protective; traits that are a basis for a secure attachment relationship [24, 26, 30]. Also, as discussed before, research shows that religious people perceive God as an actual person rather than a fictional image, and that praying towards God activates brain regions that are responsible for active interpersonal interactions, enabling people to generate an internal representation about the God [43, 44]. Finally, the fact that God is perceived as a stronger and wiser entity, often even omnipotent, omnipresent and omniscient, is promoting the theory of God as an attachment figure. Based on these statements, various experiments prove the God image to be an adequate attachment figure [25].

Based hereupon, one of the theories that Granqvist et al. developed is the correspondence pathway of attachment to God. This pathway is based upon the (at least partial) continuity of an individual’s experienced attachment relationship across time. According to this theory, a securely attached individual is expected to view God as security supporting, an avoidant attached individual is likely to turn to atheism or agnosticism, and an anxiously attached individual is likely to have an all-consuming, clingy relationship with God. Furthermore, the correspondence hypothesis supports the claim that the religious beliefs of people who are securely attached reflect the religious level of their attachment figures while insecurely attached individuals are less likely to adopt their attachment figure’s religious behaviour [21].

So, how would this theory influence the behaviour of the developed computational model? According to the theory discussed above, and individual’s religiousness and relationship to their personal God(-image) is related to their attachment style and orientation. In the model, this is represented by the ownership states and goal state. Some examples of this are given below:

1. An individual that is securely attached and has religious parents also has a secure attachment with their God-image, and shows a religiousness similar to that of their parents. The Self-ownership and God-ownership of this individual will be about the same strength, the individual has a balanced relationship to God. The goal state of earning love from God is a balanced, medium strength: the individual does want to be close and loved by God, but is not dependent on it. Because of this balance the goal state is not necessarily fulfilled or really big, like described in the previous case, so the religiousness of the individual does not fluctuate a lot.
2. An individual that has an avoidant attachment will have atheist or agnostic tendencies, and has a distant and inaccessible relationship with God. They will be hardly influenced by religious sources, e.g. ${\text{ws}}_{{God,action,a_{i} }}$, ${\text{ws}}_{{God,emotion,b_{i} }}$ and ${\text{ws}}_{{God,image_{i} }}$, and also ws_*prayer,s*_, and the Self-ownership is a lot stronger than the God-ownership, because this individual does not consider God to be influencing their life. This person also does not have a goal to earn love from God.
3. An individual that has an anxious attachment possibly has an all-consuming, grasping relationship with God, with the individual needy for the love and acceptance of the God-image. This can possibly lead to more religious behaviour as follows: First, the individual is more susceptible for religious sources, e.g. ${\text{ws}}_{{God,action,a_{i} }}$, ${\text{ws}}_{{God,emotion,b_{i} }}$ and ${\text{ws}}_{{God,image_{i} }}$, and also ws_*prayer,s*_. Moreover, the connection from God’s actions and emotions, e.g. ${\text{srs}}_{{God,a_{i} }}$ and ${\text{srs}}_{{God,b_{i} }}$ is strong, because the insecurely attached individual aspires to earn God’s love through acting in a religious manner. Also, the individual has a very strong goal state of earning love from God, and this way the individual’s behaviour is also influenced. Finally, the Self-ownership state of the individual is low in relation to the God-ownership state, because the God-image has a big influence in the life of the individual.

#### Input from prayer

Besides input from text and caregivers, another influence on the God-image is through prayer, or active communication with God. As summarized by [4], prayer can be understood as a mental activity and a form of communication with God. From the mentalizing perspective, as introduced in “Literature overview” section [43], praying is a mentalizing activity that transcends the self-other dichotomy between the Self and the God-image, and an interaction between the more personal, subjective experiences of the individual and the more objective religious, dogmatic reality that is found in religious texts and such. This way, prayer brings together both the cognitive and emotional part that creates an individual’s God image [1]. This two-sided aspect also comes forward in the study by [10] that was mentioned in “Literature overview” section, where participants either had to think about or pray for a person that angered them. The people that had to pray for the person that angered them, reported less anger and aggression than the people that had to think about that person. From the mentalizing perspective, as explained by Schaap-Jonker [43], this difference can be explained by those brain systems which become active during prayer. While thinking mainly activates brain systems in the left prefrontal cortex, prayer, as mentalizing, uses both cortical brain circuits, but also subcortical and limbic systems; combining cognitive and affective parts of the brain. This combination during prayer can lead to the activation of both an empathic view on the other, in combination with taking into account the situation, considerations and intentions of the other. This combination of the emotional and rational part during mentalizing may result in a more accepting attitude and a lower level of anger and aggression. Furthermore, the study of Schjoedt et al. [44], which was also briefly mentioned before, showed that prayer activates brain regions that are responsible for social interaction. It seems like praying individuals imagine the God they pray to as a real person, and mentalize about God, making prayer two-way traffic with the praying individuals expecting God to be influenced by their prayer in some way.

To summarize, both the doctrinal knowledge that an individual receives about God, and the individual’s character, upbringing and so forth, create a personal, internal God-image that the individual perceives as a real person, and with whom the individual interacts. In the computation model, the God-image is represented by the following process. The generation of the God-image happens through the links between the external input (World states) to the sensor states, and in the links from the sensor states to the sensory representations of the God-image. Then, the God-image influences the behaviour and emotions of the individual through the links from the sensory representations of the God-image to the ownership states, goal fulfillment state, and the preparation states.

As described above, the individual imagines God as a person with intentions and mind states [26]. In the developed model, the God-image (including images of God’s actions and emotions) is constructed by three different kinds of input, namely input about God’s emotions (mind states), actions that God performs (or intentions), and about the God image in general. This input can come from many sources, for example, religious texts or education from parents, or from prayer. The generation of the God-image from the input is modelled by the links from the world states to the sensor states (including the sensor state of the prayer, representing hearing of a prayer of someone else or of oneself), and from the sensor states to the sensory representation states of the (general) God-image, God actions and God emotions. Furthermore, while an individual’s own prayer can influence the God-image via an external connection, the individual’s prayer can also influence that individual’s God-image via an internal connection, based on links from the preparation state for the prayer to the sensory representation states for actions and emotions of the God-image and the general God-image; e.g. if an individual prays to make God happy, the emotion of his God-image might become happier (depending on the individual’s beliefs).

Part of the God-image is represented by the (adaptive) connection weights within the God-image model, partly representing the individual’s characteristics, and which may be influenced by the external input as well through Hebbian learning. These parts result in a personal God-image consisting of the individual’s sensory representation of the God-image, the individual’s sensory representation of God’s actions, the individual’s sensory representation of God’s emotions, and the weights of the connections between these three states. The conceptual representation of the model is graphically depicted in Fig. 1. In this representation, circles represent states and arrows represent processes. The dotted arrows represent Hebbian learning connections, which will be explained below. The processes that are internal are depicted inside the green box, external processes are outside the box, and the interaction between the two on the boundary.

The subscript *i* represents the difference between empathetic and dis-empathic behaviour and emotion.

An overview of the connections (the arrows) and their weights that were defined for the model can be found in Table 2, which is based on Fig. 1, and Tables 3 and 4. The first and second columns represent the states that have connections between them. The third column represents the weight of the connections between the states in the first and second columns, in respective order. The weight values depend on the specific values for the variables in the model, and on the person. In general, a weight has a value between zero and one, only *ω*_*X*_ has a value below one, this is an inhibiting connection. However, the underlined weights in the table are connections that can be learned by the person. This Hebbian learning process is explained in “A person with a disempathic God-image” section. The fourth column is the Local Properties which will be referred to in “A person with a disempathic God-image” section.

**Table 2:** Overview of connections and weights used between states in the model

From states	To state	*ω*	From states	To state	*ω*
${\text{ws}}_{{Godaction \, a_{i} }}$	${\text{ss}}_{{Godaction \, a_{i} }}$	*ω*_1_	${\text{srs}}_{{God,image_{i} }}$ ${\text{srs}}_{{God,b_{i} }}$ ${\text{srs}}_{{God,a_{i} }}$ gs_*h*_	${\text{ps}}_{{s_{i} }}$	*ω*_13a_ *ω*_13b_ *ω*_13c_ *ω*_13d_
${\text{ws}}_{{Godimage_{i} }}$	${\text{ss}}_{{Godimage_{i} }}$	*ω*_2_			
${\text{ws}}_{{Godemotion \, b_{i} }}$	${\text{ss}}_{{Godemotion \, b_{i} }}$	*ω*_3_			
${\text{ws}}_{{prayer \, s_{i} }}$	${\text{ss}}_{{s_{i} }}$	*ω*_4_			
${\text{ws}}_{{emotion \, d_{i} }}$	${\text{ss}}_{{d_{i} }}$	*ω*_5_	gfs_*h*_ gs_*h*_	gs_*h*_	*ω*_14a_ *ω*_14b_
${\text{ss}}_{{d_{i} }}$	${\text{srs}}_{{d_{i} }}$	*ω*_6_			
${\text{ss}}_{{Godaction \, a_{i} }}$ ${\text{srs}}_{{God,image_{i} }}$ ${\text{srs}}_{{God,b_{i} }}$ ${\text{ps}}_{{s_{i} }}$	${\text{srs}}_{{God,a_{i} }}$	*ω*_7a_ *ω*_7b_ *ω*_7c_ *ω*_7d_	${\text{srs}}_{{God,a_{i} }}$ ${\text{srs}}_{{God,image_{i} }}$ ${\text{srs}}_{{God,b_{i} }}$	gfs_*h*_	*ω*_15a_ *ω*_15b_ *ω*_15c_
			${\text{ps}}_{{d_{i} }}$ ${\text{srs}}_{{d_{i} }}$	${\text{fs}}_{{d_{i} }}$	*ω*_16a_ *ω*_16b_
${\text{ss}}_{{Godimage_{i} }}$ ss_*s*_ ps_*s*_	${\text{srs}}_{{God,image_{i} }}$	*ω*_8a_ *ω*_8b_ *ω*_8c_	${\text{srs}}_{{God,a_{i} }}$ srs_*e*_ fs_*d*_	${\text{os}}_{{Self,c_{i} ,d_{i} ,e_{i} }}$	*ω*_17a_ *ω*_17b_ *ω*_17c_
${\text{ss}}_{{Godemotion \, b_{i} }}$ ${\text{srs}}_{{God,image_{i} }}$ ${\text{srs}}_{{God,a_{i} }}$ ${\text{ps}}_{{s_{i} }}$	${\text{srs}}_{{God,b_{i} }}$	*ω*_9a_ *ω*_9b_ *ω*_9c_ *ω*_9d_	${\text{srs}}_{{God,a_{i} }}$ ${\text{srs}}_{{God,image_{i} }}$	${\text{os}}_{{God,a_{i} ,e_{i} }}$	*ω*_18a_ *ω*_18b_
			${\text{srs}}_{{God,b_{i} }}$ srs_*e*_	${\text{os}}_{{Self,d{}_{i},e_{i} }}$	*ω*_19a_ *ω*_19b_
${\text{ps}}_{{c_{i} }}$	${\text{srs}}_{{e_{i} }}$	*ω*_10_	${\text{srs}}_{{God,image_{i} }}$ ${\text{srs}}_{{God,b_{i} }}$	${\text{os}}_{{God,b{}_{i},e_{i} }}$	*ω*_20a_ *ω*_20b_
${\text{os}}_{{Self,c_{i} ,d_{i} ,e_{i} }}$ ${\text{os}}_{{God,a_{i} ,e_{i} }}$ ${\text{srs}}_{{God,a_{i} }}$ ${\text{fs}}_{{d_{i} }}$ gs_*h*_	${\text{ps}}_{{c_{i} }}$	*ω*_11a_ *ω*_11b_ *ω*_11c_ *ω*_11d_ *ω*_11e_	${\text{os}}_{{God,a_{i} ,e_{i} }}$ ${\text{os}}_{{Self,c_{i} ,d_{i} ,e_{i} }}$ ${\text{ps}}_{{c_{i} }}$	${\text{es}}_{{c_{i} }}$	*ω*_21a_ *ω*_21b_ *ω*_21c_
${\text{os}}_{{Self,d_{i} ,e_{i} }}$ ${\text{os}}_{{God,b{}_{i},e_{i} }}$ ${\text{srs}}_{{God,b_{i} }}$ ${\text{srs}}_{{e_{i} }}$ ${\text{fs}}_{{d_{i} }}$ gs_*h*_	${\text{ps}}_{{d_{i} }}$	*ω*_12a_ *ω*_12b_ *ω*_12c_ *ω*_12d_ *ω*_12e_ *ω*_12f_	${\text{os}}_{{Self,d_{i} ,e_{i} }}$ ${\text{os}}_{{God,b{}_{i},e_{i} }}$ ${\text{ps}}_{{d_{i} }}$	${\text{es}}_{{d_{i} }}$	*ω*_22a_ *ω*_22b_ *ω*_22c_
			${\text{ps}}_{{s_{i} }}$	${\text{es}}_{{s_{i} }}$	*ω*_23_
			${\text{es}}_{{s_{i} }}$	${\text{ws}}_{{s_{i} }}$	*ω*_24_
			${\text{es}}_{{d_{i} }}$	${\text{ws}}_{{d_{i} }}$	*ω*_25_

**Table 3:** Description of abbreviations used in the model

Abbreviation	Description
ws_*W*_ *W*: *prayer s*_*i*_; *God*-*image*_*i*_; *Godaction a*_*i*_; *Godemotion b*_*i*_; *d*_*i*_	A world state *W*
ss_*W*_ *W*: *prayer s*_*i*_; *God*-*image*_*i*_; *Godaction a*_*i*_; *Godemotion b*_*i*_; *d*_*i*_	Sensor state for *W*
srs_*X*_ *X*: *God,image*_*i*_; *God, a*_*i*_; *God, b*_*i*_; *d*_*i*_; *e*_*i*_	Sensory representation state for *X*
ps_*Y*_ *Y*: *c*_*i*_*, d*_*i*_*, s*_*i*_	Preparation state for executing *Y*
${\text{os}}_{{God,a{}_{i},e_{i} }}$	Ownership state for God of action *a*_*i*_ and effect *e*_*i*_
${\text{os}}_{{Self,c_{i} ,b_{i} ,e_{i} }}$	Ownership state for the Self of action *c*_*i*_, effect *e*_*i*_ and emotion *b*_*i*_
${\text{os}}_{{God,b{}_{i},e_{i} }}$	Ownership state for God of emotion *b*_*i*_ and effect *e*_*i*_
${\text{os}}_{{Self,d{}_{i},e_{i} }}$	Ownership state for the Self of emotion *d*_*i*_ and effect *e*_*i*_
es_*Y*_ *Y*: *c*_*i*_; *b*_*i*_; *s*_*i*_	Execution state for *Y*: executing *c*_*i*_ or expressing *b*_*i*_ or *s*_*i*_
${\text{fs}}_{{b_{i} }}$	Feeling state for *b*_*i*_
gs_*h*_	Goal state for *h*
gfs_*h*_	Goal fulfillment state for *h*

**Table 4:** Description of variables used in the model

Variable	Description
*a*_*i*_ *i*: 1;2	Either an empathic action for the God image (*a*_1_) or a dis-empathic action (*a*_2_)
*b*_*i*_	An emotion of the God-image (associated to action effect), either an empathic emotion (*b*_1_) or a dis-empathic emotion (*b*_2_)
*c*_*i*_	A behaviour option for the Self, related to action *a*_*i*_ of the God-image, either empathic behaviour (*c*_1_) or dis-empathic behaviour (*c*_2_)
*d*_*i*_	A feeling of the Self related to emotion *b*_i_ of the God-image, (associated to action effect *e*_i_); either an empathic feeling (*d*_1_) or a dis-empathic feeling (*d*_2_)
*e*_*i*_	The predicted effect of a behaviour
*h*	goal that an individual can have
*s*_*i*_	A prayer that an individual can execute, either internal or externally expressed, and either conscious or unconscious

### From conceptual to numerical representation of the model

This section describes the process of numerical formalization of the model presented in “Mirror neurons and internal simulation”, “Action ownership states for God and Self”, and “The God-image” sections. This formalization was used to implement the model in Python to perform simulations. According to the adopted Network-Oriented Modelling approach, a graphical conceptual representation displays nodes for *states* and arrows for *connections* indicating causal impacts from one state to another, and includes some additional labels for states and connections, so that it becomes a labelled graph:

- Connection weights ω_***X,Y***_ for each connection from state *X* to state *Y.*
- Combination functions c_***Y***_**(..)** to aggregate multiple impacts for each state *Y.*
- Speed factors ***η***_***Y***_ for speed of change for each state *Y.*

To choose combination functions, a number of standard options is available; e.g. [47, 48]. The conceptual representation of a temporal–causal network model can be transformed in a systematic or even automated manner into the following numerical representation of the model [30, 31]; here the variable *t* indicates a time point; it varies over the real numbers. Based on a combination function and the connection weights

1 $${\mathbf{aggimpact}}_{\varvec{Y}} (t) \, = \, \varvec{c}_{\varvec{Y}} \left( {\varvec{\omega}_{{\varvec{X}_{{\mathbf{1}}} ,\varvec{Y}}} X_{1} \left( t \right), \ldots ,\omega_{{\varvec{X}_{\varvec{k}} ,\varvec{Y}}} X_{k} \left( t \right)} \right)$$

is the *aggregated impact* of the network on *Y* at *t*. This is used to provide the following *difference* and *differential equation* for each state *Y*:

2 $$\begin{aligned} Y(t + \Delta t) &= Y(t) +\varvec{\eta}_{Y} \left[ {{\mathbf{aggimpact}}_{\varvec{Y}} (t) - Y(t)} \right]\Delta t \\ & = Y(t) +\varvec{\eta}_{\varvec{Y}} \left[ {\varvec{c}_{\varvec{Y}} \left( {\varvec{\omega}_{{\varvec{X}_{{\mathbf{1}}} ,\varvec{Y}}} X_{1} \left( t \right), \, \ldots ,\varvec{\omega}_{{\varvec{X}_{\varvec{k}} ,\varvec{Y}}} X_{k} \left( t \right)} \right) \, - Y\left( t \right)} \right]\Delta t, \hfill \\ \end{aligned}$$

3 $$\begin{aligned} \text{d}Y(t)/\text{d}t &=\varvec{\eta}_{\varvec{Y}} \left[ {{\mathbf{aggimpact}}_{\varvec{Y}} (t) - Y(t)} \right] \hfill \\ & =\varvec{\eta}_{\varvec{Y}} \left[ {\varvec{c}_{\varvec{Y}} \left( {\varvec{\omega}_{{\varvec{X}_{{\mathbf{1}}} ,\varvec{Y}}} X_{1} \left( t \right), \ldots ,\varvec{\omega}_{{\varvec{X}_{\varvec{k}} ,\varvec{Y}}} X_{k} \left( t \right)} \right) \, - Y\left( t \right)} \right]. \hfill \\ \end{aligned}$$

These numerical representations (2) and (3) can be used for mathematical and computational analysis and simulation. In the model presented here, for all states for the combination function the *advanced logistic sum combination function* **alogistic**_*σ*,*τ*_**(***…***)** is used [30, 31]:

4 $$\begin{aligned} {\mathbf{c}}_{Y} (V_{ 1} , \, \ldots V_{k} ) &= {\mathbf{alogistic}}_{\sigma ,\tau } (V_{ 1} , \, \ldots ,V_{k} ) \hfill \\ & = \left( {\frac{1}{{1 + {\text{e}}^{{ - \sigma \left( { V_{1} + \cdots + V_{k} - \tau } \right)}} }} - \frac{1}{{1 + {\text{e}}^{\sigma \tau } }}} \right) \, (1 + {\text{e}}^{ - \sigma \tau } ). \hfill \\ \end{aligned}$$

Here *σ* is a *steepness* parameter and *τ* a *threshold* parameter. The advanced logistic sum combination function (4) has the property that activation levels 0 are mapped to 0 and it keeps values below 1. When the value of the right hand side expression given above is < 0, the value 0 is assigned to **alogistic**_*σ*,*τ*_**(***V*_1_*,…, V*_*k*_**)**.

In cases of adaptive networks in which some or all of the connection weights *ω*_*X,Y*_ are dynamic, for a numerical representations dynamic connection weights also get a time argument: *ω*_*X,Y*_(*t*). To model their dynamics, the dynamic connection weights are described by a difference or differential equation for Hebbian learning, which also can be based on a combination function and speed factor as above; for more details, see [47, 48]. In the current network model, learning mechanism was included for the connection strengths of the adaptive connections from ${\text{srs}}_{{God,image_{i} }}$ to ${\text{srs}}_{{God,a_{i} }}$, from ${\text{srs}}_{{God,image_{i} }}$ to ${\text{srs}}_{{God,b_{i} }}$, from ${\text{srs}}_{{God,a_{i} }}$ to ${\text{srs}}_{{God,b_{i} }}$, and from ${\text{srs}}_{{God,b_{i} }}$ to ${\text{srs}}_{{God,a_{i} }}$; see the dotted lines in Fig. 1. This learning mechanism is based on the Hebbian learning principle introduced by Donald Hebb [14]. Different interpretations of Hebbian learning exist, either based on causality-based learning [29] or simultaneity-based learning; e.g. [6, 7, 49]. In this model, the latter simultaneity-based learning approach is used. This approach is based on the principle that strengthening of a connection between neurons over time may take place when both nodes are often active simultaneously: ‘neurons that fire together, wire together’ [44]. In the model, the weight *ω*_*X*,*Y*_ of an adaptive connection from state *X* to state *Y* is updated after time step Δ*t* using a learning rate *η*_H_ > 0 and extinction rate *ζ*_H_ ≥ 0, and the activation levels *X(t)* and *X(t)* of the states *X* and *Y*. This is modelled as follows (see also [13], p. 406):

5 $$\omega_{X,Y} \left( {t + \Delta t} \right) = \omega_{X,Y} \left( t \right) + \left[ {\eta_{H} X\left( t \right)Y(t)\left( {1 - \omega_{X,Y} \left( t \right)} \right) - \zeta_{H} \omega_{X,Y} \left( t \right)} \right]\Delta t.$$

The weight *ω*_*X*,*Y*_ has a maximal strength of 1; the factor 1 − *ω*_*X*,*Y*_(*t*) keeps *ω*_*X*,*Y*_ below 1.

## Simulation scenarios

As discussed, the computational model was implemented in Python to perform simulations and study the influence of religion on human empathy and dis-empathy. Simulations have focused on six possible scenarios based on the literature. For each scenario, relevant parameter values are chosen to simulate the behaviour described in the literature and to test the influence on empathic or dis-empathic behaviour. For most of the states in the implemented model, two instances are used: the empathic (indicated with subscript 1 in the figures) and the dis-empathic instance (indicated with subscript 2 in the figures). Through adapting the connections relating to those two instances, the degree of empathy of dis-empathy of the God-image or individual can be varied. In the first scenario, in “A person with a neutral God-image” section a person with a neutral God-image is presented, to form some sort of a ‘neutral’ beginning. Then, a person with an empathic God-image is presented in “A person with an empathic God-image” section, and one with a dis-empathic God-image in “A person with a disempathic God-image” section, to compare the influence of the relation of an individual to God on their behaviour and emotions. After that, a person with autism is simulated, to test whether these kinds of disorders have impact on how religion influences behaviour. Then, in the last two sections, two ‘extreme’ scenarios are simulated, one for a person that is atheist in “A person with autism” section, to find whether an individual that is not religious is still influenced in their behaviour, and finally “A person that is atheist” section is a simulation of an individual with fundamentalist tendencies, and how this influences their behaviour towards others. For each scenario, ∆*t* was chosen 0.25, the total number of time steps 500, and the speed factor of all states 0.17. The extinction and learning rates for the adaptive connections are all 0.5. A certain combination of parameters within a person could lead to fundamentalist tendencies. For example, if a person has both an anxious attachment relationship with the God image, a dis-empathic God-image, and a lot of divinity and dis-empathic related external influence about God, this could form behaviour that is considered fundamentalist.

### A person with a neutral God-image

**Fig. 2:**
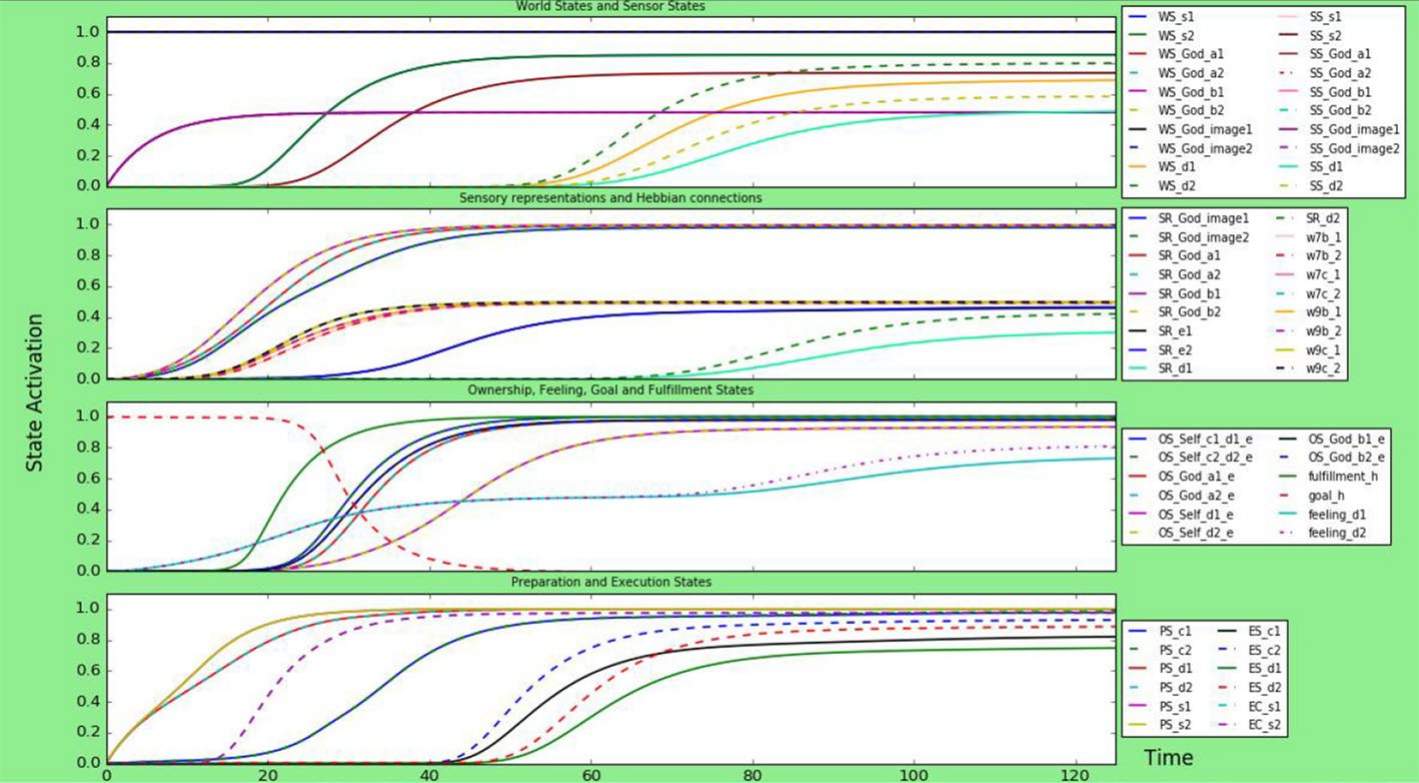
Simulation scenario for a person with a neutral God-image, meaning that the empathic and dis-empathic parameters are the same strength. The person executes dis-empathic and empathic actions and emotions with more or less the same activation strength and around the same time

This scenario simulates a person with a neutral God-image. Neutral in this context means that the connections to the empathic part and dis-empathic parts of the God-image, and external input on both of these, are the same strength. All connections are of medium strength; around 0.55, 0.7, and 0.8, besides some connections from states that are not split between empathic or dis-empathic, such as the goal state. The results can be found in Fig. 2.

### A person with an empathic God-image

**Fig. 3:**
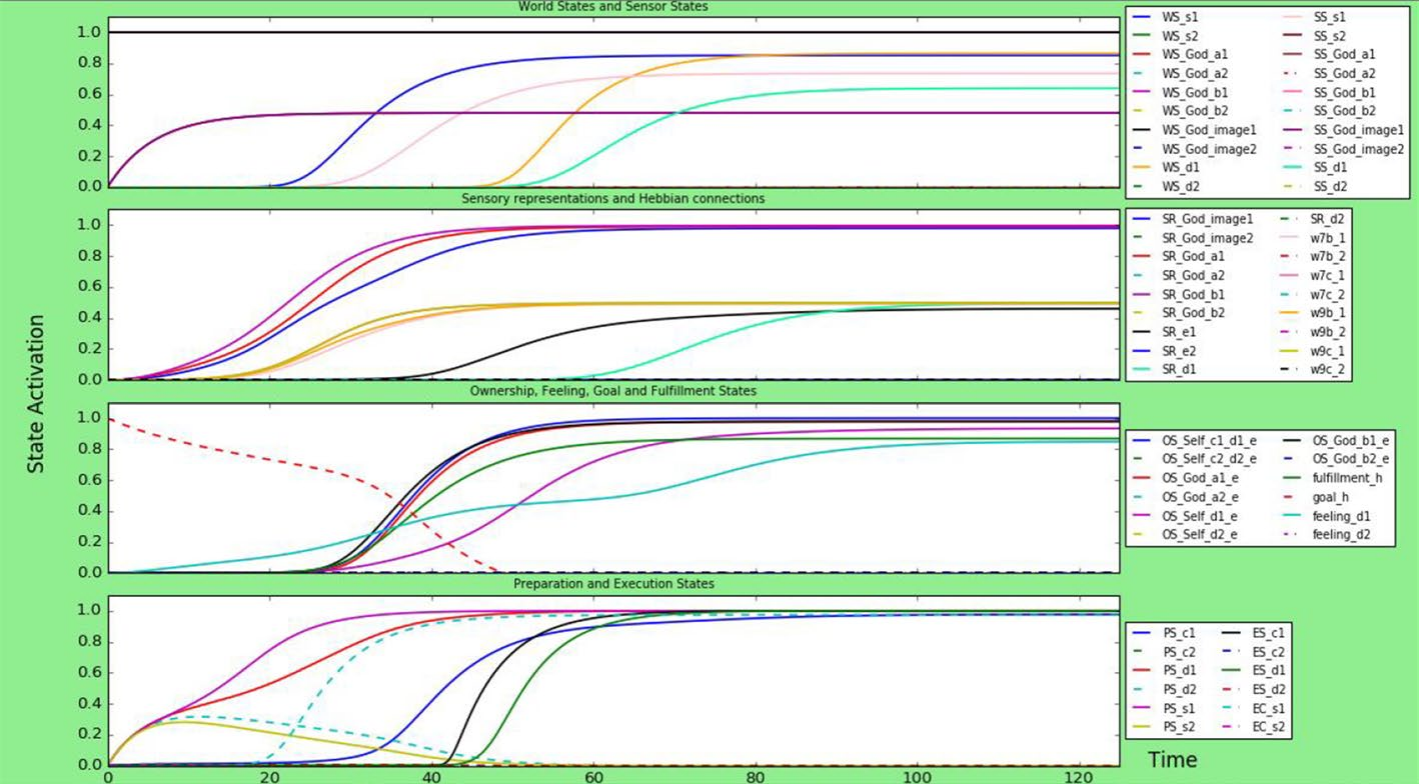
Simulation scenario for a person with an empathic God-image, meaning that the empathic part of the model has stronger parameters than the dis-empathic part. The person executes only empathic behaviour in contrast to dis-empathic behaviour

The second scenario is that of a person with an empathic God-image, which means a stronger empathic part and external influence on the God-image. In this person, connections to the empathic part of the individual’s God-image are stronger than those to the dis-empathic part. The ‘empathic’ connections are around 0.8, while the dis-empathic connections are around 0.1. The result can be found in Fig. 3. In contrast to “A person with a neutral God-image” section, only the empathic sensor states start developing while the dis-empathic ones remain inactive. This is the same for the sensory representations and feeling state: only the empathic ones become active, while the dis-empathic ones remain inactive. The goal fulfillment becomes activated at a later time point than in “A person with a neutral God-image” section, namely around time point 30, causing the goal state to decrease slower. Finally, the preparation states for all behaviour and emotion execution start increasing from time point 0, also the dis-empathic ones. However, the dis-empathic preparation states do not become more active than 0.3 and decrease again after time point 10, while the empathic preparation states keep increasing until activation 0.9/1.0. Finally, the execution of empathic behaviour and expression of empathic emotions starts at time point 40, reaching an equilibrium around time point 70 at activation value 1.0, while the dis-empathic behaviour and emotions are never executed.

### A person with a dis-empathic God-image

**Fig. 4:**
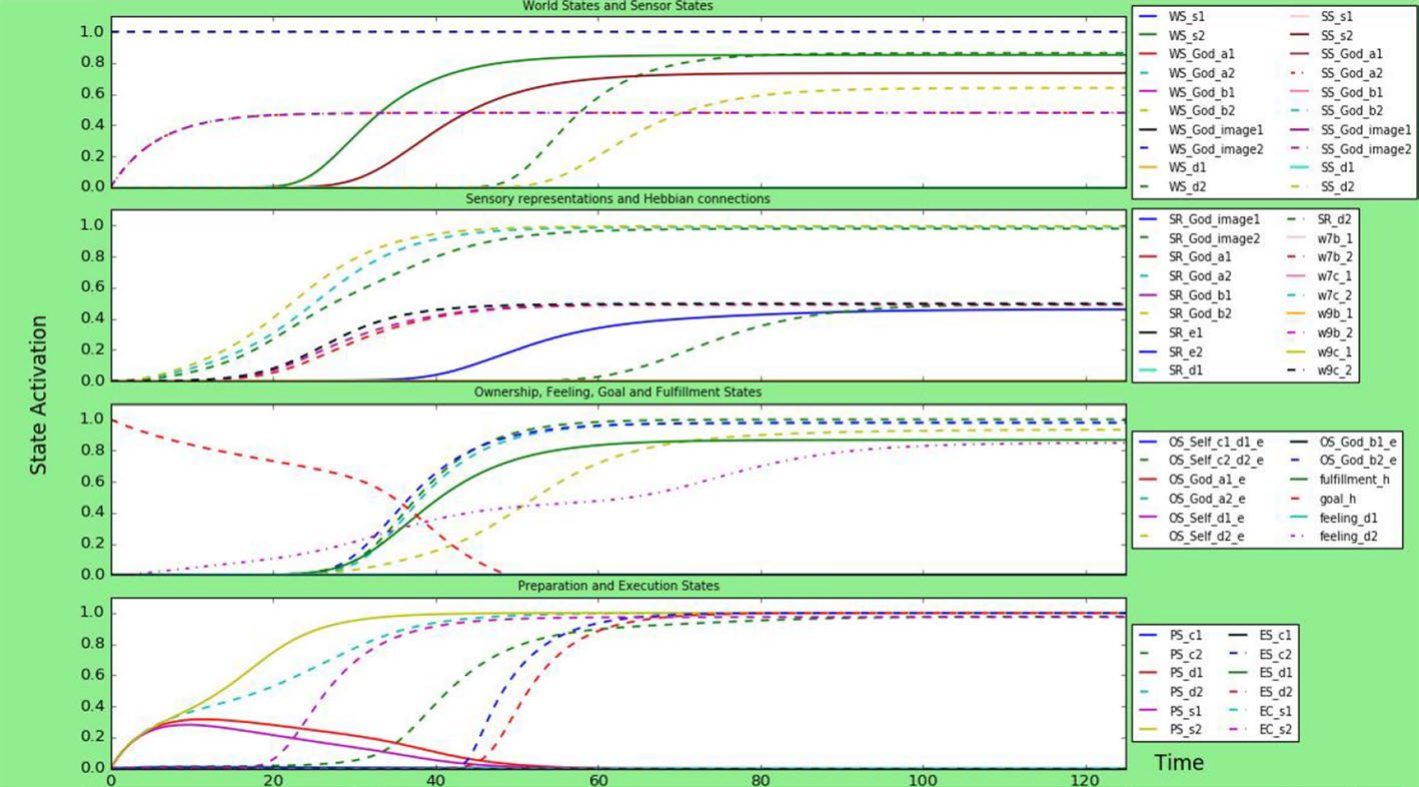
Simulation scenario for a person with a dis-empathic God-image, meaning that the dis-empathic part of the model has stronger parameters than the empathic part. The person executes only dis-empathic behaviour in contrast to empathic behaviour

The third simulation is that of a person with a dis-empathic God-image. In this person, the dis-empathic part of the individual’s God-image overrules the empathic part. The ‘empathic’ connections are around 0.8, while the dis-empathic connections are around 0.1 (mirroring the simulation of “A person with an empathic God-image” section). The result can be found in Fig. 4. In contrast to “A person with an empathic God-image” section, only the dis-empathic sensor states start developing while the empathic ones remain inactive. This is the same for the sensory representations and feeling state: only the dis-empathic ones become active, while the empathic ones remain inactive.

Again, the goal fulfillment becomes activated on a later time point than in “A person with a neutral God-image” section, namely around time point 30, causing the goal state to decrease slower. Finally, as in “A person with an empathic God-image” section, the preparation states for all behaviour and emotion execution start increasing from time point 0, also the empathic ones. However, the empathic preparation states do not become more active than 0.3 and decrease again after time point 10, while the dis-empathic preparation states keep increasing until activation 0.9/1.0. Finally, the execution of dis-empathic behaviour and expression of empathic emotions starts at time point 40, reaching an equilibrium around time point 70 at activation value 1.0, while the empathic behaviour and emotions are never executed.

### A person with autism

**Fig. 5:**
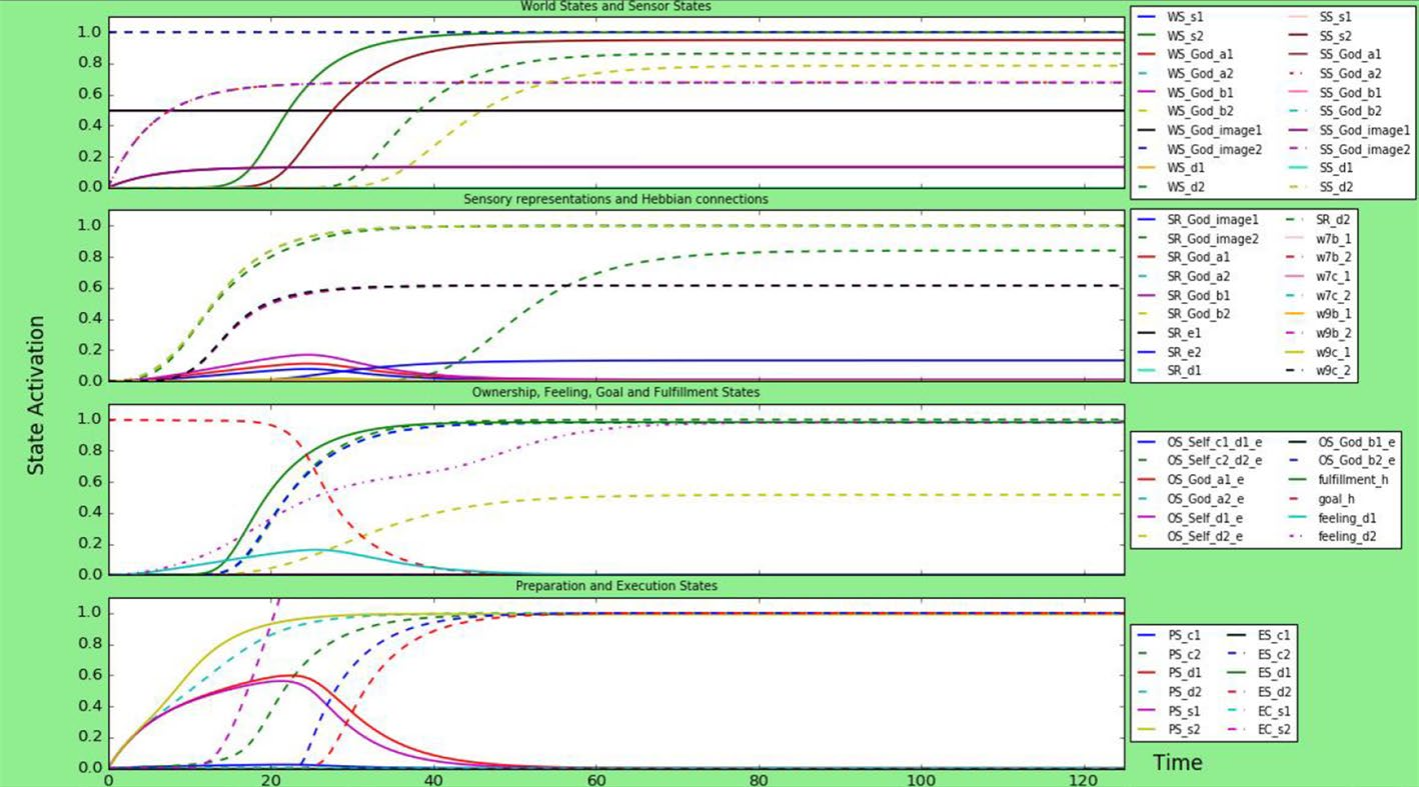
Simulation scenario for a person with ASD and impairments in the social domain. The mirroring mechanism and internal simulation mechanism have lower strength, and the person has a dis-empathic God-image. Only dis-empathic actions and emotions are executed, lower Self-ownership is generated

As discussed in “The temporal–causal network model” section, mentalizing and mirror neurons are important aspects of an individual’s religious belief and God-image. However, there are parts of the populations where these processes work in atypical ways, for example, in people with autism [17, 50, 55]. This could mean that individuals on certain parts of the autism spectrum have a different religious experience than other individuals. Recently, various studies have been done on this topic [36, 42], showing that there are differences in religious belief and God-image among autistic individuals. For example, Schaap-Jonker found that individuals with Autism Spectrum Disorders had a God-image with fewer positive and more negative characteristics than the God-image of individuals without Autism Spectrum Disorders. This effect was especially stronger among individuals with Autism Spectrum Disorders that experienced impairments in the social domain, who felt anxiety towards their God-image and perceived God as punishing. On the other hand, religious salience predicted positive characteristics of the image of God of individuals with Autism Spectrum Disorders. In the computational model, this can be simulated as follows:

- A lower connection strength (around 0.3) of the mirroring mechanism, which are the connection from the sensory representations of the God image, God emotions and God actions to the preparation states of the individual’s actions or emotions.
- A lower connection strength (around 0.3) of the internal simulation/mentalizing mechanism, which are the connections from the preparation states for an individual’s actions to the sensory representations for the effects of those actions.
- For an individual with Autism Spectrum Disorders that experiences difficulties in the social domain: stronger negative external influence, and stronger links from dis-empathic God actions and emotions: the dis-empathic connections are around 1.0, while the empathic connections are around 0.8. The negative external influences ${\text{ws}}_{{Godaction\_a_{2} }}$, ${\text{ws}}_{{Godemotion\_b_{2} }}$ and ${\text{ws}}_{{God,image_{2} }}$ are 1.0, while the positive external influences, ${\text{ws}}_{{Godaction\_a_{1} }}$, ${\text{ws}}_{{Godemotion\_b_{1} }}$ and ${\text{ws}}_{{God,image_{1} }}$ are 0.5. The results can be found in Fig. 5.

Thus, from time point 0, the negative world states are 1, while the positive ones are 0.5, the goal state gs_*h*_ starts at 1.0. This causes the positive sensor states to increase to much lower activation values than the negative ones, although they all start at the same time points as in “A person with a neutral God-image”, “A person with an empathic God-image” and “A person with a disempathic God-image” sections. Then, the sensory representations for the negative God image start developing similar to previous scenarios, although they are steeper, but the sensory representations for the positive God image only reach a very low activity value, around 0.1. Also, the ownership states for the negative actions and emotions develop ‘normally’, although also these are steeper than before, while the ownership states for the negative actions and emotions do not develop at all anymore. However, the self-ownership state ${\text{os}}_{{Self,d_{2} ,e_{2} }}$ for the negative emotion only reaches half the activation value of the God-ownership state ${\text{os}}_{{God,d_{2} ,e_{2} }}$ for the negative emotion.

At time point 0, the preparation states for the dis-empathic behaviour and emotions develop as normal, although again more steep, while the preparation states for the empathic behaviour and emotions only reach activation value 0.58 before it starts decreasing at time point 30. Finally, the execution of dis-empathic behaviour and emotions reaches the same activation value as in the other scenarios, around 1.0, although the equilibrium is reached at time point 50 instead of 70 (in previous scenarios). The empathic behaviour and emotions are not executed at all.

### A person that is atheist

**Fig. 6:**
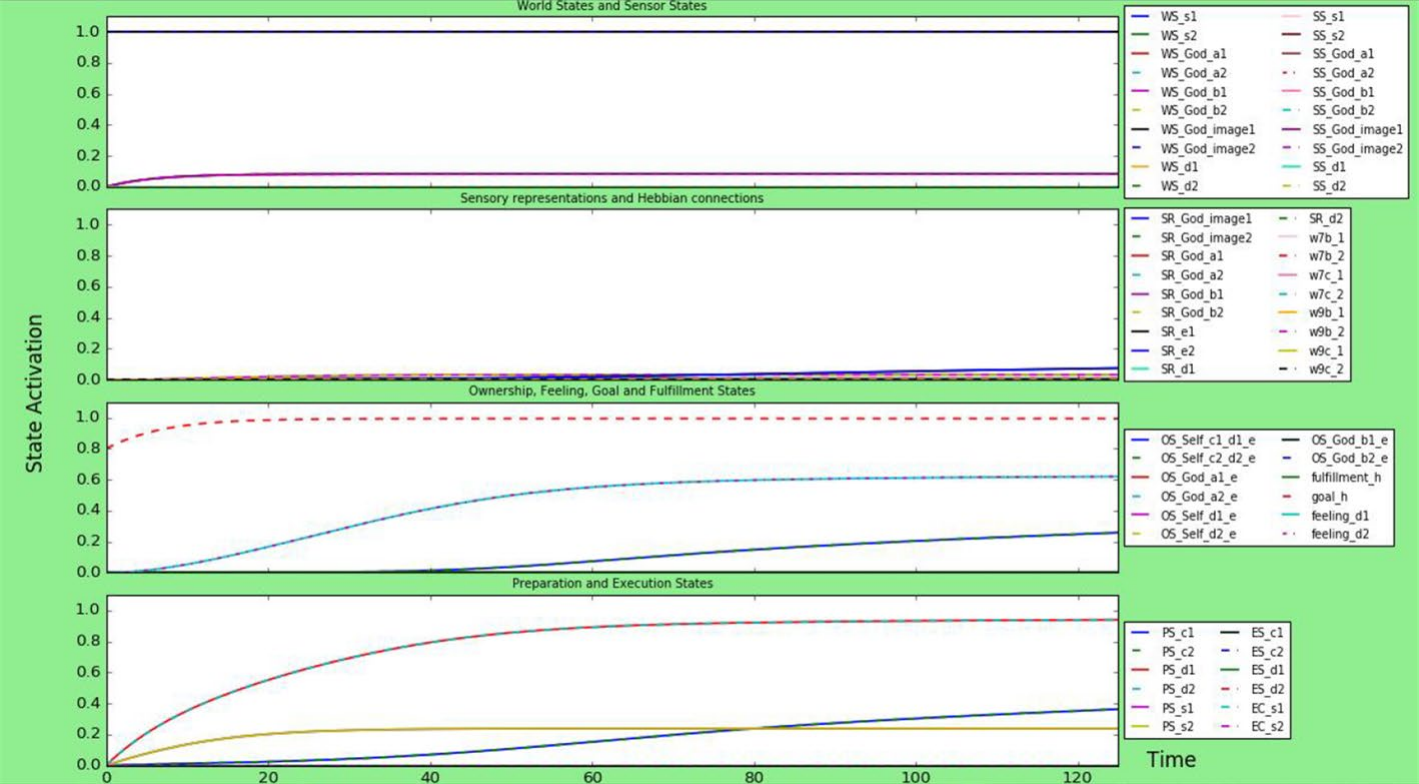
Simulation scenario for an atheist person, with low connection strengths between world states and sensor states, low connections to God-ownership, high connections to Self-ownership and a lower goal state. The atheist does not really show religious behaviour, although there is a preparation state for prayer

As discussed in “The temporal–causal network model” section, an avoidant attachment relationship can possibly lead to an individual with atheist or agnostic tendencies, and a distant relationship to God. This person will be hardly influenced by religious sources. For example, ${\text{ws}}_{{God,action\_a_{i} }}$, and ${\text{ws}}_{{God,image_{i} }}$, and also ${\text{ws}}_{{prayer,s_{i} }}$, and the connections to the Self-ownership are a lot stronger (around 1.0) than those to the God-ownership (around 0.3), because this individual does not consider God to be influencing their life. However, as studied by Lindeman et al. [32], even atheists become emotionally aroused when thinking of God, hence why the values mentioned above are not zero. Finally, the atheists’ goal state is lower than previous scenarios, 0.8 instead of 1, because this individual does not aspire to be close to God. As can be seen by briefly looking at Fig. 6, the atheist individual is hardly influenced by religion: All the world states, sensor states and sensory representations are either not or very weakly activated. The goal state is higher because it is not fulfilled, and both feeling states are also increasing until around 0.6. Finally, the atheist person does develop emotion preparation states with a relatively high activation level, around 0.9, which matches the result of Lindeman et al. [32], and also some activation for the execution of prayer, although this is not very high: maybe even atheists sometimes say an (unconscious) little prayer. However, with this model it is difficult to judge the behaviour of atheists, since the model does not represent the individual’s actions besides the influence from the God-image, and atheists are only lightly influenced by the God-image. For example, [45] discussed that the social behaviour of atheists and theists are motivated by different social cues: where atheists social behaviour is triggered by God-related cues, atheist’s social behaviour is triggered by secular institutions, such as courts and police. Still, it is interesting to see the difference in influence of the God-image on atheists in comparison to religious individuals.

### A person with fundamentalist tendencies

**Fig. 7:**
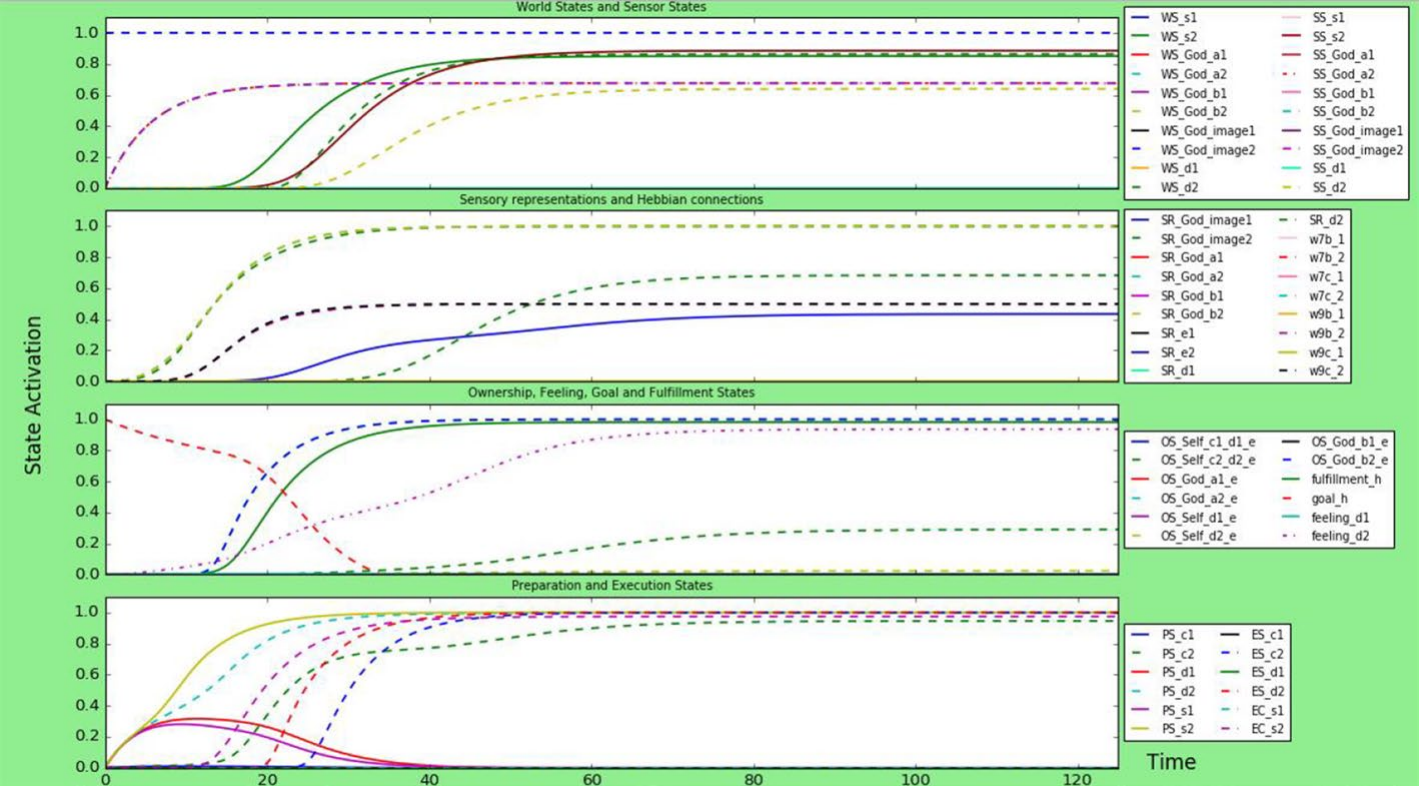
Simulation scenario for a person with fundamentalist tendencies, meaning that connection strengths to the Self-ownership are very low while the God-ownership is high, there is low, connection strengths to the effect prediction is low and the person has a dis-empathic God-image. The person strongly executes dis-empathic behaviour, no empathic behaviour, and develops no Self-ownership

A certain combination of parameters within a person could lead to fundamentalist tendencies, as discussed in “The temporal–causal network model” section. If a person has both an anxious attachment relationship with the God image, a dis-empathic God-image, and a lot of divinity and dis-empathic related external influence about God, this could form behaviour that is considered fundamentalist. This scenario tries to simulate this fundamentalist behaviour by making the dis-empathic connections in the model higher than the empathic ones (1.0 versus 0.1), making the God ownership states higher than the Self-ownership state (God-ownership for empathic behaviour 0.8, for dis-empathic behaviour 0.3, Self-ownership 0.1) and strong links to from dis-empathic God-image to the preparation states (1.0) and from preparation states to execution states (1.0). The result can be found in Fig. 7.

Main differences with a scenario with a person with a dis-empathic God-image are as follows: the fundamentalist person does not, or barely, develop Self-ownership of its actions; the fundamentalist person does have a lower activation level of the prediction of the effects of his actions: ${\text{srs}}_{{e_{i} }}$; the dis-empathic behaviour of the fundamentalist person reaches the same activation level, but reaches this level faster than the person with just a dis-empathic God-image.

## Discussion and conclusion

In this paper, the influence of religion on human empathy and dis-empathy was studied. First of all, an extensive literature study was done regarding all the processes are related to religion and human behaviour, specifically towards others. The relevant theory was then used to design a conceptual representation of a temporal–causal network model that captures the process of how religion influences human behaviour, for example, the religion-related external input that an individual receives, the way this external input is then processes and generates a personal God-image, and how this God-image influences the individual’s behaviour and emotions. The behaviour and emotions of both the God-image and the individual were distinguished in empathic and dis-empathic. Although (informally expressed) theories exist and are referred in the different sections above, a formalized computational model for them was never designed, as far as the authors know; so, comparison with other computational models is difficult.

The developed conceptual representation was then formalized into the numerical representation and this was implemented in Python. With this implemented network model, scenarios based on the relevant literature were addressed to simulate the influence of religion on human empathy and dis-empathy, to answer the question asked in the beginning. For example, scenarios were simulated for a person with an empathic or dis-empathic God-image, persons with atheist or fundamentalist tendencies, or persons with Autism Spectrum Disorders. It was shown how a person mirrors the empathy or dis-empathy in the actions and emotions of the God-image, depending on the situation of a person. First of all, it was shown how external (religious) influences have impact on an individual’s God-image. Input regarding a dis-empathic God created a dis-empathic God-image, while input regarding an empathic God generated an empathic God-image. Furthermore, the God-image strongly influenced the empathic or dis-empathic behaviour and emotions of the religious individual. An empathic God-image led to empathic actions and emotions, while a dis-empathic God-image led to dis-empathic actions and emotions. However, there were more aspects that influenced this. For example, the ownership and mirroring process: persons with a very low Self-ownership can show more fundamentalist tendencies.

Although the simulations and the model in general show some interesting results, it is difficult to provide a final answer on what the influence of religion on human empathy and dis-empathy is. While the model does represent important aspects of the domain, and is a good basis for an answer, there are still many things to improve. For example, the model only reflected the influence of the God-image on the behaviour of the individual, not that of other persons or more specific non-addressed characteristics of the person itself. Therefore, the process of literature study, developing a conceptual model, formalizing it and simulating is an iterative one, where adaptations can be made all the time to match the real world situation as much as possible while preserving the abstractness that is required of a computational model.
